# Public Concern about Air Pollution and Related Health Outcomes on Social Media in China: An Analysis of Data from Sina Weibo (Chinese Twitter) and Air Monitoring Stations

**DOI:** 10.3390/ijerph192316115

**Published:** 2022-12-01

**Authors:** Binbin Ye, Padmaja Krishnan, Shiguo Jia

**Affiliations:** 1 College of Chinese Language and Culture, Jinan University, Guangzhou 510610, China; 2 Division of Engineering, New York University Abu Dhabi, Abu Dhabi P.O. Box 129188, United Arab Emirates; 3 School of Atmospheric Sciences, Sun Yat-Sen University and Southern Marine Science and Engineering Guangdong Laboratory (Zhuhai), Zhuhai 519082, China; 4 Guangdong Provincial Field Observation and Research Station for Climate Environment and Air Quality Change in the Pearl River Estuary, Guangzhou 510275, China; 5 Guangdong Province Key Laboratory for Climate Change and Natural Disaster Studies, Sun Yat-Sen University, Guangzhou 510275, China

**Keywords:** air pollution, public health concern, social media, Sina Weibo, data mining

## Abstract

To understand the temporal variation, spatial distribution and factors influencing the public’s sensitivity to air pollution in China, this study collected air pollution data from 2210 air pollution monitoring sites from around China and used keyword-based filtering to identify individual messages related to air pollution and health on Sina Weibo during 2017–2021. By analyzing correlations between concentrations of air pollutants (PM_2.5_, PM_10_, CO, NO_2_, O_3_ and SO_2_) and related microblogs (air-pollution-related and health-related), it was found that the public is most sensitive to changes in PM_2.5_ concentration from the perspectives of both China as a whole and individual provinces. Correlations between air pollution and related microblogs were also stronger when and where air quality was worse, and they were also affected by socioeconomic factors such as population, economic conditions and education. Based on the results of these correlation analyses, scientists can survey public concern about air pollution and related health outcomes on social media in real time across the country and the government can formulate air quality management measures that are aligned to public sensitivities.

## 1. Introduction

Air pollution, one of the leading causes of public health concern, is gaining increased attention, especially in developing countries. Studies have shown that the air pollution crisis could have physical and psychological impacts on the population [1]. Ambient air pollution may cause respiratory and cardiovascular diseases and can also result in increased emergency department visits and daily mortality rates [2,3]. Air pollution is also associated with a high risk of developing chronic degenerative diseases in children [4]. Long-term exposure to a high concentration of PM_2.5_ contributes to genotoxicity, mutagenicity and cancer [5,6]. According to the World Health Organization (WHO), 4.6 million people die from illnesses directly related to poor air quality each year [7], and 4000 preventable deaths have been attributed to air pollution in China [8,9]. More recent studies have also shown that air pollution could increase susceptibility to COVID-19 and the prognosis of patients affected by COVID-19 and suggested measures that can be used to reduce its spread [10,11]. In addition to physical health effects, air pollution can also lower one’s overall mental being, as studies have shown that it leads to a lower “expressed happiness” [12], serious psychological distress, and depression [13], and it may even increase the risk of suicide [14].

In recent years, the Chinese government has formulated many countermeasures, such as the Three-Year Action Plan on Defending the Blue Sky, to achieve the strategic transformation from emission control to air quality management and tackling air pollution issues in China [15,16]. Emissions of SO_2_ and NO_X_ and concentrations of PM_2.5_ and PM_10_ were shown to have significantly decreased in most cities in China, though PM concentrations through China were still found to be higher than the recommended long-term and short-term air quality guidelines (AQG) levels by the WHO [17]. Moreover, the control measures implemented during the COVID-19 outbreak curtailed personal mobility and economic activities and resulted in decline of NO_2_ and SO_2_ concentrations in urban areas of China [18,19]. However, for the successful implementation of air pollution control measures, it is necessary for the government to understand and track the public’s response to the pollution measures. There is a limited understanding of public concern about air pollution and related health outcomes, especially under the spatiotemporal variations of people‘s exposure to air pollution in China [20]. Social media postings by individuals are a viable tool that can be harnessed to understand people’s response to air pollution measures. Regarded as a “social sensor”, social media can provide data that can be mined and analyzed [21,22]. Collecting data from Sina Weibo, which is the largest microblogging service platform in China, can aid researchers understand public concern about air pollution and related health outcomes, as well as help researchers further examine the relationship between pollutant concentrations and related microblogs.

Previous studies analyzed data for shorter durations of one to two years and examined air-pollution-related tweets and air pollution data represented by PM_2.5_ concentration or air quality index (AQI) values. Mei et al. [23] collected microblogs containing the word “霾 (haze)” and AQI information for one month in 2013 to establish a machine learning model that could estimate AQI from social media messages. As research into health threats posed by air pollution deepened, studies focused on tweets related to the health outcomes of air pollution. Wang et al. [24] manually filtered messages on Sina Weibo in 2013 using a set of health-related terms from the Chinese medical dictionary and air-pollution-related terms, and they found that these messages had a strong correlation with PM_2.5_ concentrations in 74 cities in China. More recent studies tried to distinguish health-related tweets from tweets simply related to air pollution and then compared them with the level of air pollution. Gurajala et al. [25] used supervised learning to identify health-related tweets among air-pollution-related tweets that were posted in London, New Delhi, and Paris from September 2015 to May 2018, and they determined that in New Delhi, which has a poor air quality, PM_2.5_ concentrations were strongly correlated with not only air-pollution-related tweets but also health-related tweets. It is difficult to evaluate the long-term variations of air pollution and related microblogs with only one or two years of data. Short-term studies cannot reveal variations of social media postings with the rapid changes in air pollution in China in recent years. Moreover, studies have shown that there are some differences in the public’s sensitivity to major air pollutants (PM_2.5_, PM_10_, CO, O_3_, SO_2_ and NO_2_) across China [26]. Therefore, a longer-duration study including data on concentrations of different pollutants and related microblogs is needed in order to understand the long-term effects of air pollution through social media postings.

The relationship between air pollution and related messages on social media is affected by air quality and socioeconomic factors. On one hand, correlations between pollutant concentrations and related microblogs would be stronger if air quality became worse. In Beijing, correlation coefficients between AQI and air-pollution-related microblogs were found to be higher in winter and spring, with the worst AQI, and lower in summer and autumn, with the best AQI [27]. On the other hand, socioeconomic factors have not been fully considered when researchers discuss the association of air pollution and social media. The effect of socioeconomic factors on the relationship between air pollution and related microblogs requires further discussion, since pollutant concentrations and perception of air pollution can be influenced by socioeconomic factors, such as population, urbanization rate, per-capita gross regional product, traffic factors, and human mobility [28,29,30]. Research has also revealed the spatial dependence effect of public health and the effects of per capita income and per capita education level on improving public health [31]. Therefore, it is crucial to examine the effect of socioeconomic factors on air pollution and related microblogs.

As the first research examining air pollution and Weibo data for a period of five years and to associate socioeconomic factors with the relationship between air pollution and related microblogs, this study utilized air pollution data from 2210 air pollution monitoring sites all over China and Weibo data collected by the Application Programing Interface (API) of Sina Weibo from 2017 to 2021 and analyzed the correlation between air-pollution-related microblogs, health-related microblogs, and concentrations of six air pollutants (PM_2.5_, PM_10_, CO, NO_2_, O_3_ and SO_2_). Specifically, this research was focused on three questions: (1) Which pollutant had the strongest correlation with air-pollution-related and health-related messages on Sina Weibo? (2) What were the temporal variation and spatial distribution of the relationship between air pollution and related microblogs during 2017–2021 in China? (3) What socioeconomic factors influenced the relationship between air pollution and related microblogs?

## 2. Materials and Methods

### 2.1. Air Pollution Data

Air pollution data were collected from an online platform monitoring and analyzing air quality (https://www.aqistudy.cn/ (accessed on 18 March 2022)). Data from 2210 air pollution monitoring sites in 74 cities of 31 Provincial Administrative Regions (PARs) in China from 1 January 2017 to 31 December 2021 were used in this study. The PARs considered in this study were 22 provinces (Anhui, Fujian, Gansu, Guangdong, Guizhou, Hainan, Hebei, Henan, Heilongjiang, Hubei, Hunan, Jilin, Jiangsu, Jiangxi, Liaoning, Qinghai, Shandong, Shanxi, Shaanxi, Sichuan, Yunnan, and Zhejiang), 5 autonomous regions (Guangxi, Inner Mongolia, Ningxia, Tibet, and Xinjiang) and 4 municipalities (Beijing, Chongqing, Shanghai, and Tianjin) and excluded Taiwan, Hong Kong and Macao since the online platform did not have their air quality data. In this research, the pollutant concentrations reported for a PAR are the average values of pollutant concentrations for all air quality monitoring stations in the PAR. Six air pollutants—PM_2.5_, PM_10_, CO, NO_2_, O_3_ and SO_2_—were analyzed in this research, and the measurement unit for pollutant concentration was μg/m^3^ (except for CO, which was measured as mg/m^3^).

### 2.2. The Weibo Data

Sina Weibo, one of the most popular social media platforms in China, referred to as the Chinese Twitter, allows users to upload microblogs or messages. The messages posted by the Sina Weibo users are called “Weibos” and have a 140-Chinese-character limit, similar to “tweets” from English language social media platform Twitter. According to the 2020 Weibo User Trends Report released by the Weibo Data Center (https://data.weibo.com/ (accessed on 5 April 2022)), the number of monthly active users of Sina Weibo reached 511 million as of September 2020.

#### 2.2.1. Data Collection

**Figure 1:**
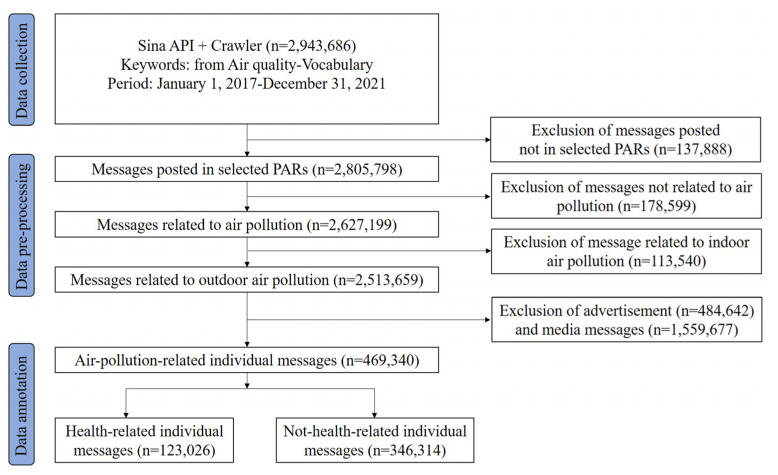
Flow diagram of data processing.

The analysis of Weibos related to air pollution was carried out through data collection, pre-processing and annotation, as shown in Figure 1. Using the API of Sina Weibo and a web crawler provided by Sina Weibo that simulates “advanced search”, the platform was searched for Weibos based on keywords and posting time, and a set of 2,943,686 social media messages related to air pollution were obtained for the period from 1 January 2017 to 31 December 2021. A list of air-pollution-related terms from the Air Quality Vocabulary promulgated by the Ministry of Environmental Protection of China in 2009 (HJ 492-2009) were selected as keywords, e.g., “空气污染” (air pollution), “空气质量” (air quality), “霾” (haze), “粉尘” (dust), “气溶胶” (aerosol) and “空气 + 排放” (air + emission). Other terms, including “PM_2.5_”, “沙尘” (sand dust), “能见度” (visibility) and “空气 + 臭” (air + smelly), were also manually added as keywords [24,32].

#### 2.2.2. Data Pre-Processing

Firstly, the places of registration of the users were restricted to the 31 PARs selected in this research to ensure that all the social media messages used in the research were posted in places with online air pollution data [27]. After filtering the messages based on the users’ registered locations, 137,888 social media messages were removed since they did not have precise information (“none”, “others” or “China”), referred to places outside China (“Japan”, “Korea”, “USA” etc.) or were not located in the selected PARs (“Hong Kong”, “Macao”, or “Taiwan”).

Secondly, a total of 178,599 microblogs not related to air pollution and 113,540 microblogs about indoor air pollution were removed as noise. A list of keywords such as “雾霾蓝” (haze blue) were used to filter out microblogs that were not about air pollution. Microblogs with the keywords “室内” (indoor) or “甲醛” (formaldehyde) were also filtered out because the focus of this research was on outdoor air pollution [27].

Thirdly, 484,642 advertisements were removed as noise based on the keywords and usernames they contained. An analysis of the microblogs revealed that most of the advertisements contained products relevant to air pollution (such as “air conditioner”, “fresh air system”, and “facial cleanser”) and terms used in online sales (such as “free shipping”, “voucher”, and “best seller”), as well as usernames with “company”, “shop” or “group”, and these were used to remove the advertisements.

Finally, media messages (*n* = 1,559,667) were filtered out since the focus of this research was on public response to air pollution rather than public agencies’ response. Moreover, after removing media messages, the remaining individual messages were found to have a stronger correlation with air pollutant concentrations [33]. Media messages released by public agencies responsible for weather forecasts often had usernames containing “天气” (weather), “气象” (meteorology), “生态” (ecology), “环境” (environment), names of cities, and keywords such as “AQI”, “优” (excellent), “良” (good), and “浓度” (concentration) [27]. Media messages probably sharing news stories also included the symbol “【】” in the headline or “报” (newspaper), “电视” (TV), or “在线” (on-line) in the username. In the end, 469,340 individual microblogs related to air pollution were used for further analysis.

As mentioned above, different keyword-based filters were used to segregate the Weibos related to air pollution. However, it needs to be verified whether the chosen Weibos were accurate in terms of their content and reliable in terms of whether they were posted by individuals. Ultimately, 2000 air-pollution-related individual messages that had undergone the filtering process were randomly selected as test samples for data pre-processing accuracy verification to manually judge whether these samples were related to air pollution and posted by individuals [24]. The test results showed that the accuracy of data pre-processing was 86.25%, which was within the margin of error. This means that the keyword-based filter used in data pre-processing was acceptable. Hence, individual messages related to air pollution after filtering constituted the dataset of Weibos that could reflect public concern about air pollution and were used in the correlation analyses between air pollution and related Weibos.

#### 2.2.3. Data Annotation

Keyword-based filtering was used to identify health-related individual messages (*n* = 123,026) among air-pollution-related individual messages (*n* = 469,340) by checking for health-related keywords. Data training was conducted to identify health-related keywords from 1843 individual messages that were randomly selected. If an individual message was related to the health outcomes of air pollution, it was manually labeled and then health-related terms were chosen from the text of this message. Messages were independently coded by two annotators, and a third annotator was invited to resolve any disagreements [24]. Finally, 177 health-related keywords were selected from individual messages relevant to air pollution. As shown in Table 1, the health-related keywords could be categorized into four types: general health-related words, body parts, prevention and treatment, and diseases and symptoms.

### 2.3. Statistical Analysis

To compare air pollution data with related individual messages on Sina Weibo, Pearson correlation analyses were employed to understand the relationship between air pollutant concentrations and the number of air-pollution-related or health-related individual messages on Sina Weibo. Air-pollution-related individual messages were denoted as APR Weibos, and health-related individual messages were designated as HR Weibos. The number of APR or HR Weibos was calculated by month because some PARs with smaller populations may have had days when no HR Weibos were posted. Accordingly, air pollutant concentrations were reported as the monthly average concentrations of PM_2.5_, PM_10_, CO, NO_2_, O_3_ and SO_2_, and they were calculated as the average values of daily air pollutant concentrations in a month. Based on the location of air pollution monitoring sites and the places of registration for Sina Weibo users, the monthly average concentrations of air pollutants were found to correspond one-to-one with the monthly APR or HR Weibos from 2017 to 2021 in the 31 selected PARs (*n* = 1860).

## 3. Results

### 3.1. General Description

#### 3.1.1. Frequency of Keywords

The primary public concerns about air pollution and related health outcomes on social media could be visualized in word clouds where the words were sized based on their frequency of occurrence in Weibos and translated from Chinese into English (see Figure S1). It is obvious that visible particulate matter led to more discussion on Sina Weibo. The top five air-pollution-related keywords were “haze”, “air quality”, “visibility”, “air pollution” and “PM_2.5_”. Haze and PM_2.5_ were commonly mentioned in the Weibos since their effect on reducing visibility was easily perceived. Furthermore, increasing risk of respiratory diseases was considered to be one of the most serious threats of air pollution to health by the public. “Disease”, “breath”, “health”, “mask” and “lung” were the most frequently used health-related keywords. Interestingly, people were not only concerned about the general health issues related to air pollution but also often mentioned the function (“breath”), protective gear (“mask”), and organ (“lung”) of the respiratory tract when they discussed air pollution.

#### 3.1.2. Spatiotemporal Characteristics of Air Pollution

**Figure 2:**
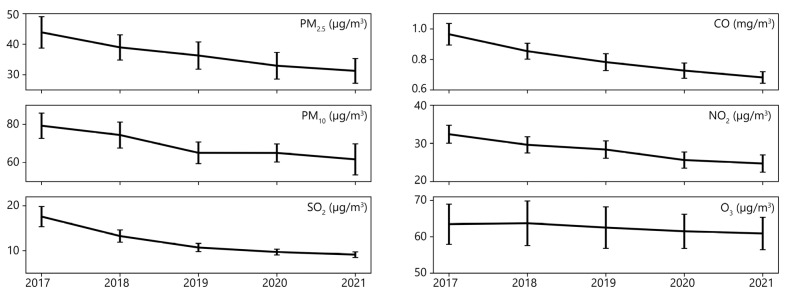
Annual average concentration of six air pollutants in 2017–2021. Error bar represents standard deviation.

Air quality in China improved from 2017 to 2021 as the annual average concentrations of air pollutants decreased (Figure 2). Annual average concentrations of PM_2.5_, PM_10_, CO, NO_2_, and SO_2_ dropped more than 20% over five years (especially the concentration of SO_2_, which dropped 48.16% in 2021 relative to 2017), though the O_3_ concentration remained at nearly the same level from 2017 to 2021. In 2021 across the 31 PARs, the annual average concentrations of PM_2.5_, PM_10_, CO, NO_2_, O_3_ and SO_2_, respectively, were 31.26 μg/m^3^, 61.63 μg/m^3^, 0.68 mg/m^3^, 24.73 μg/m^3^, 60.92 μg/m^3^ and 9.12 μg/m^3^. The concentrations for the gaseous pollutants were all lower than the national concentration limit prescribed by the Environmental Air Quality Standards (GB3095-2012), and the annual average concentration of both particulate pollutants, PM_2.5_ and PM_10_, met the recommended long-term interim target 1 set by the WHO global air quality guidelines.

**Figure 3:**
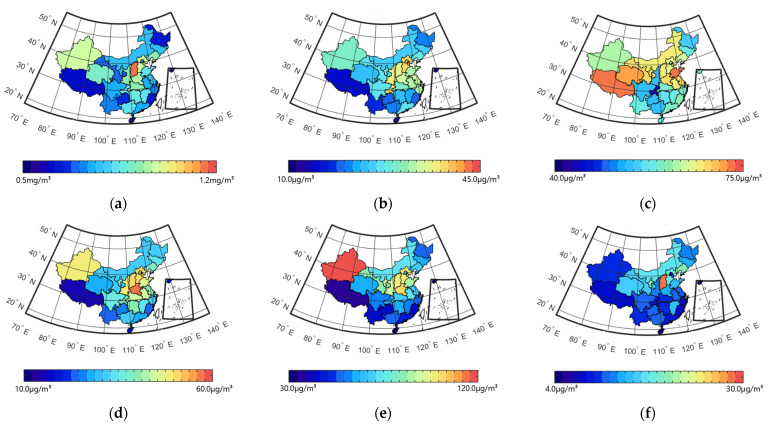
Average concentrations of air pollutants during 2017–2021 in 31 PARs: (**a**) CO; (**b**) NO_2_; (**c**) O_3_; (**d**) PM_2.5_; (**e**) PM_10_; (**f**) SO_2_.

As shown in Figure 3, the average concentrations of PM_2.5_, PM_10_, CO, NO_2_, O_3_ and SO_2_ for the period of 2017–2021 decreased from north to south. Air pollution was extremely serious in the Beijing–Tianjin–Hebei region, Central Plains, Shandong Peninsula urban agglomerations, and Xinjiang in Northwest China. Regions that had excellent air quality during the study period were the Yunnan–Guizhou Plateau and Tibet Province in Southwest China (with sparse population and less industrialization) and southeastern coastal areas of China that experience windy and rainy weather that aids pollutant diffusion. Annual average concentrations of particulate matter, PM_2.5_ and PM_10_, were extremely high in the Beijing–Tianjin–Hebei region and surrounding areas, including Henan and Shanxi in Central Plains, which have large populations and a heavy industry, and Xinjiang in Northwest China, which is affected by frequent dust storms [34]. The spatial distribution of CO was similar to that of PM_2.5_ and PM_10,_ and high concentrations of NO_2_ were observed in the Yangtze River Delta because of the dense population and economical activities in the region [35]. In addition, the SO_2_ concentration in northern areas was higher than in southern areas due to the burning of more coal for winter heating [36], and Shanxi (in Central Plains) recorded the highest SO_2_ concentrations among the PARs, mainly due to coal burning [34].

The highest O_3_ concentrations were observed in Shandong and attributed to the large population (101,650,000 in 2020) in Shandong Peninsula’s urban agglomerations. However, the spatial distribution of O_3_ was not entirely consistent with other air pollutants. Tibet (in Southwest China) had good air quality, but its annual average O_3_ concentration was extremely high. Tibet is located in the Qinghai–Tibet Plateau with an average altitude of over 4000 m. At this altitude, photochemical reactions, vertical mixing, and the downward transport of stratospheric air mass occur, thus raising O_3_ concentrations [37]. For the same reason, Qinghai also experiences a high annual average concentration of O_3_.

#### 3.1.3. Spatiotemporal Characteristics of Weibo

**Figure 4:**
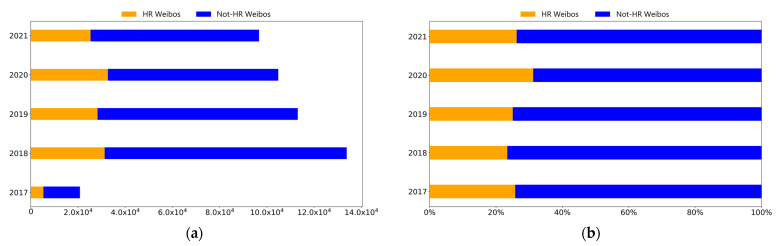
(**a**) Number and (**b**) percentage of HR Weibos among APR Weibos in 2017–2021 (the number of APR Weibos is the sum of HR and not-HR Weibos).

APR Weibos could be divided into two types: HR Weibos, which revealed public concern about air pollution and related health outcomes, and not-HR Weibos, which reflected public concern about air pollution but not specifically on health outcomes. The annual variations of APR and HR Weibos are shown in Figure 4.

The number of APR Weibos dramatically increased from 2017 to 2018 and then showed a downward trend from 2018 to 2021 (see Figure 4a). In 2017, only 20,845 APR Weibos were posted by individuals, but in 2018, the figure rose to 133,836, which was 6.42 times the number of Weibos posted in 2017. The number of APR Weibos then slowly declined year by year to 96,700 in 2021. The huge number of APR Weibos in 2018 could have been due to the implementation of the Three-Year Action Plan on Defending the Blue Sky by the Chinese government in 2018 to decrease air pollution and ensure blue skies, and it also may have been the reason for the significant improvement in the air quality in China from 2018 to 2021 (see Figure 2).

The number of HR Weibos reached a peak in 2020, as there were 32,696 HR Weibos among the 104,845 APR Weibos. Health issues, in general, garnered great attention from the public in 2020 due to COVID-19, which emerged in December 2019 and was announced as a pandemic in March 2020 by the WHO. Interestingly, the percentage of HR Weibos among APR Weibos remained relatively close during the entire period (see Figure 4b), ranging from 23.40% (in 2018) to 31.19% (in 2020).

Table 2 shows the spatial patterns of Weibo data distribution and the Pearson correlation coefficients between monthly APR and HR Weibos in each PAR during 2017–2021 (*n* = 60).

First of all, the spatial distribution of APR or HR Weibo postings and the locations of Weibo users were consistent with the economic development of the considered regions. PARs with the largest number of APR and HR Weibo postings were located in Beijing (in the Beijing–Tianjin–Hebei region), Guangdong (in the Pearl River Delta), and Shanghai, Jiangsu and Zhejiang (in the Yangtze River Delta). As the Weibo User Trends Report in 2020 showed, the places of registration for Weibo users were often located in the Beijing–Tianjin–Hebei region, the Pearl River Delta, and the Yangtze River Delta, which are home to large populations and have more developed economies. This could be the reason why people in those PARs posted a large amount of APR and HR Weibos.

Furthermore, correlations between APR and HR Weibos were significant (*p* < 0.05) and strong (*r* > 0.6), not only overall (China) but also individually for most PARs. A strong and significant correlation existed between APR and HR Weibos in 31 PARs together (*n* = 1860), with a correlation coefficient of 0.901 and *p* < 0.01. Correlations between APR and HR Weibos in each PAR (*n* = 60) were found to be statistically significant (*p* < 0.01) and strong (*r* > 0.6), as shown in Table 2, except for Guizhou (*r* = 0.340). That means if the number of APR Weibo postings increased, the number of HR Weibo postings would likely correspondingly increase in each PAR. The percentage of HR Weibos among APR Weibos was relatively close in all 31 PARs, ranging from 20.21% to 32.04%. Due to the strong relation between APR and HR Weibos, the two are discussed together in the following sections.

### 3.2. Influence of Pollutants on Air Pollution and Health Related Weibos

#### 3.2.1. Public’s Sensitivity to Different Pollutants

The number of APR or HR Weibo postings was found to significantly increase whenever the concentrations of PM_2.5_, PM_10_ and NO_2_ increased in China during 2017–2021. Pearson correlation analyses revealed that correlations between APR Weibos and concentrations of PM_2.5_ (*r* = 0.162), PM_10_ (*r* = 0.080), and NO_2_ (*r* = 0.223) were statistically significant (*p* < 0.05) and positive (*r* > 0), as were correlations between HR Weibos and concentrations of PM_2.5_ (*r* = 0.111) and NO_2_ (*r* = 0.152).

For individual PARs, there were six PARs showing significant (*p* < 0.05) and positive (*r* > 0) correlation between APR Weibos and PM_2.5_ or PM_10_ concentrations. Additionally, there were three PARs with a significant (*p* < 0.05) and positive (*r* > 0) correlation between HR Weibos and PM_2.5_ concentration, and there were five PARs with a significant (*p* < 0.05) and positive (*r* > 0) correlation between HR Weibos and PM_10_ concentration. However, there were no or only one PAR with significant (*p* < 0.05) and positive (*r* > 0) correlations between concentrations of other pollutants (CO, NO_2_, O_3_ and SO_2_) and APR or HR Weibos in 2017–2021, which was less than those for PM_2.5_ and PM_10_ concentrations. The number of PARs with significant (*p* < 0.05) and positive (*r* > 0) correlations was not much during 2017–2021 because the number of APR and HR Weibos sharply rose in 2018 while pollutant concentrations remained relatively stable from 2017 to 2021. To ensure the accuracy of the statistical results, correlations between pollutant concentrations and APR or HR Weibos were separately analyzed for each year from 2017 to 2021, as further discussed in Section 3.3.

Correlations between PM_2.5_ or PM_10_ concentrations and related Weibos were more statistically significant than those for other air pollutants, not only in China as a whole but also in each PAR during 2017–2021. The reasons why particulate matter, PM_2.5_ and PM_10_, easily led to public concern about air pollution and health issues are elucidated in the following paragraphs. Firstly, increases in particulate matter, PM_2.5_ and PM_10_, may prompt more discussion about air pollution on Sina Weibo since they are more easily perceived by naked eyes than gaseous pollutants (CO, NO_2_, O_3_ and SO_2_). There was a significant congruence of particles less than 10 μm in diameter and perceived air pollution, as revealed by a China Social Survey [38]. By analyzing the PM and meteorological data from 1988 to 2012, it was found that fine PM, such as PM_2.5_, had a key influence on visibility in the Yangtze River Delta in China [39]. This is also the reason why “haze”, “visibility” and “PM_2.5_” became three of the top five keywords in APR Weibos with the highest occurrence (in Section 3.1.1).

Moreover, people tend to associate easily perceived pollutants with causes of diseases. PM_2.5_, particles with a diameter of 2.5 μm or less, are considered to be a serious threat to health because they can not only go deep into the lungs and cause respiratory diseases but may also contain carcinogenic constituents that can increase the risk of pulmonary diseases such as emphysema, lung cancer, and nasal cancer [40,41,42]. Other pollutants can also lead to diseases. For instance, exposure to NO_2_ raises the risk of respiratory disease [43], long-term O_3_ exposure is associated with death from respiratory disease [44], and SO_2_ pollution may trigger ischemic cardiac events [45]. However, people may not associate diseases with invisible air pollutants because it is difficult to accurately understand the responses of sensory organs in the human body to variations in the concentrations of gaseous pollutants.

To examine the relationship between pollutant concentrations and related Weibos in 2017–2021, correlations between concentrations of CO, NO_2_, O_3_, PM_2.5_, PM_10_, SO_2_ and APR or HR Weibos were analyzed for each year during the studied period (*n* = 372). As shown in Table 3, correlations between NO_2_ concentration, PM_2.5_ concentration, and related Weibos (including APR and HR Weibos) were significant (*p* < 0.05) and positive (*r* > 0) for each year during the period from 2017 to 2021.

Among the six air pollutants evaluated in this study, only PM_2.5_ concentration had a significant and positive correlation with APR or HR Weibos both in China, and in most PARs, and the relationship remained stable during the period from 2017 to 2021. In other words, the public was shown to be most sensitive to changes in PM_2.5_ concentration. It is worth noting that even PM_10_ concentrations were not significantly correlated with related Weibos in each year of 2017–2021. As Table 3 shows, the correlations between PM_2.5_ concentration and HR Weibos were significant (*p* < 0.05) and positive (*r* > 0) in each year of 2017–2021, while PM_10_ concentrations were only significantly correlated with HR Weibos in 2017 and 2021. Fine particles (PM_2.5_) can penetrate deep into the alveolar region of humans, while coarse particles (PM_2.5–10_) are mainly deposited in tracheobronchial airways; therefore, fine particles are more harmful to humans than coarse particles [46]. This may be the reason why people tend to associate health issues more with PM_2.5_ than PM_10_ on social media.

#### 3.2.2. Factors Influencing Relationship between Air Pollution and Related Weibos

Correlations between pollutant concentrations and APR or HR Weibos were analyzed in each PAR during 2017–2021 (*n* = 60), and the plots of correlation coefficients against the annual average concentrations of NO_2_, PM_2.5_ and PM_10_ are shown in Figure S2. CO, O_3_ and SO_2_ are not included in the scatter plots because the R^2^ values in those models were less than 0.1. Additionally, correlations between APR or HR Weibos and concentrations of CO, O_3_ and SO_2_ were not significant and positive in China during 2017–2021 (see Section 3.2.1).

As Figure S2 shows, the correlation between air pollutants and related Weibos was stronger in PARs with higher annual average pollutant concentrations, as seen with NO_2_, PM_2.5_ and PM_10_. This means that residents in a PAR with poor air quality have higher chances to post more APR or HR Weibos as the pollutant concentrations increase. This was also seen in previous studies that mentioned that poor air quality in a region had a higher chance to trigger people to directly complain on social media and post more Weibos related to air pollution [23]. Furthermore, the Air Discussion Index (ADI), built using terms in Weibos most associated with varying air quality conditions, was found to be strongly correlated with the measured PM_2.5_ in Beijing with poor air quality. However, in Guangzhou, Shanghai and Chengdu, with relatively lower PM_2.5_ concentrations, the correlation between ADI and PM_2.5_ concentration was not so strong [47].

### 3.3. Air Pollution and Related Weibos: Temporal Variation

#### 3.3.1. PARs with Significant and Positive Correlations

**Figure 5:**
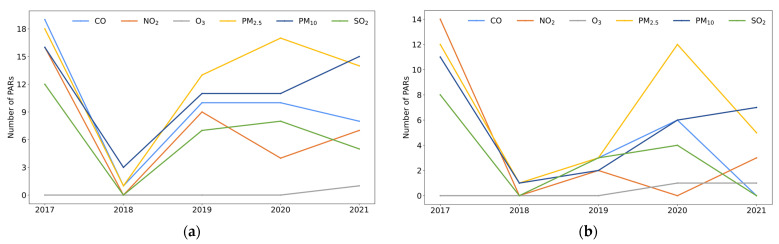
Number of PARs with a significant and positive correlation between air pollution and related Weibos in each year of 2017–2021 for: (**a**) APR Weibos; (**b**) HR Weibos.

Figure 5 shows the number of PARs with significant (*p* < 0.05) and positive (*r* > 0) correlations for each of the six pollutants (PM_2.5_, PM_10_, CO, NO_2_, O_3_, and SO_2_) and APR or HR Weibos for each year from 2017 to 2021.

First of all, the number of PARs with a significant and positive correlation between pollutant concentrations (except for O_3_) and APR or HR Weibos in 2017 was more than in any other year from 2018 to 2021, with the lowest number of PARs recorded in 2018 (see Figure 5). As in Figure 2, annual average concentrations of pollutants showed a downward trend and air quality in China improved during the period 2017–2021 with the implementation of the Three-Year Action Plan on Defending the Blue Sky from 2018 [34]. It is possible that with a decrease in air pollution, fewer people perceived air pollution as a major issue and posted Weibos about it. It was found that lower correlation coefficients of the AQI and related Weibos were linked to a period with the lowest value and the narrowest range of the AQI in a study in Beijing [27]. Moreover, due to the commencement of the Three-Year Action Plan on Defending the Blue Sky in 2018, more Weibos related to general air quality issues were posted than Weibos focusing on changes in air pollutant concentrations, and this could be the reason for the low correlation between pollutant concentrations and APR Weibos in 2018.

Furthermore, the number of PARs with a significant and positive correlation between pollutant concentrations and HR Weibos peaked twice, once in 2017 and a second time in 2020 or 2021, as shown in Figure 5b. For example, there were 12 PARs with a significant and positive correlation between PM_2.5_ concentration and HR Weibos in 2017; this figure dropped to only one PAR with a significant and positive correlation in 2018 and three PARs in 2019. However, it increased back to 12 PARs in 2020 before decreasing to five PARs in 2021. The number of PARs with a significant and positive correlation was highest in 2017 because that year also recorded the highest pollutant concentrations, and there were two reasons for the second largest number of PARs with a significant and positive correlation between pollutant concentrations and HR Weibos in 2020 or 2021. One possibility is that the emergence of the COVID-19 pandemic in 2020 heightened public concern over health issues and resulted in increases in the number and percentage of HR Weibos in 2020 (see Figure 4). Another possibility is that the relationship between air pollution and related Weibos was not only related to pollutant concentrations but could have also been affected by some socioeconomic factors that are further discussed in the following section.

#### 3.3.2. Socioeconomic Factors Influencing APR or HR Weibo Postings

Socioeconomic factors such as population, economic conditions, and education are associated with public concern about air pollution and related health outcomes on social media. Based on a correlation analysis between APR or HR Weibo posts and socioeconomic factors (see Table 4) in 31 PARs during 2017–2020 (*n* = 1488), the correlations between APR Weibos and population (*r* = 0.331), GDP per capita (*r* = 0.555), and schooling years per capita (*r* = 0.464) were found to be statistically significant at *p* < 0.01, and there were also significant (*p* < 0.01) correlations between HR Weibos and population (*r* = 0.354), GDP per capita (*r* = 0.533), and schooling years per capita (*r* = 0.434). In other words, the PARs with larger populations, higher GDP per capita, and longer schooling years per capita have more educated and high-income Weibo users who might pay more attention to air pollution and related health outcomes and therefore post more related Weibos.

Population, economic conditions (represented by GDP per capita), and education (represented by schooling years per capita) could influence public concern about air pollution and health outcomes on social media for two possible reasons. Firstly, the socioeconomic factors of a PAR may influence the attributes and number of users on social media, which can determine how many Weibos are posted. Users on social media vary in age, gender, income, education and individual behavior [48,49], and a previous study showed that such models used to predict social media posts may not be applicable to regions with extremely low social media user populations [23]. Secondly, the socioeconomic factors of a PAR are also known to affect air pollution and related health outcomes there. Economic conditions affect PM_2.5_ concentration by influencing the availability of transportation facilities and construction [50], and groups with low socioeconomic status or communities with low-income populations may have more air pollution exposure [51,52]. It is also well-acknowledged that health-related problems are common in low-income countries since 92% of pollution-related deaths occur in low-income and middle-income countries [53].

To further examine whether socioeconomic factors influenced the relationship between pollutant concentrations and related Weibos, correlations between correlation coefficients of pollutant concentrations and APR or HR Weibos in each PAR in each year of 2017–2020 and socioeconomic factors in 31 PARs during 2017–2020 were analyzed, as shown in Table 5 (*n* = 124). Since the public is most sensitive to PM_2.5_ in China, only correlation coefficients between PM_2.5_ concentration and APR Weibos (denoted as *r*_1_) and correlation coefficients between PM_2.5_ concentration and HR Weibos (denoted as *r*_2_) are shown in Table 5.

Education and economic conditions were found to be key socioeconomic factors influencing relationship between pollutants and related Weibos. As shown in Table 5, schooling years per capita showed significant correlations with *r*_1_ (*p* = 0.022) and *r*_2_ (*p* = 0.007). In other words, if residents had a higher level of education, correlations between PM_2.5_ concentration and related Weibos were stronger. Moreover, the correlations between schooling years and GDP per capita were significant (*p* < 0.01) and strong (*r* = 0.680) because the education level of residents and economic development are closely related. Public concern about air pollution and related health outcomes was directly associated with the education and income level of residents in a PAR. The social characteristics of individuals, such as area-level economic characteristics represented by the percent of the population in poverty and individual-level psychological characteristics including knowledge, were found to be key factors that influenced the public perception of air pollution and related health concerns in a study in the Kansas City metropolitan area [54]. People who are rich and well-educated may pay more attention to air pollution [55], while air quality is rated worse where minorities and poverty are concentrated, showing that the perception of air pollution is affected by neighborhood socioeconomic position [56].

### 3.4. Air Pollution and Related Weibos: Spatial Distribution

#### 3.4.1. Distribution of PARs with Number of Years in Significant and Positive Correlation

The number of PARs with significant and positive correlation between PM_2.5_ concentrations and related weibos was higher than that between other pollutant concentrations and related weibos during the study period (2017–2021). Furthermore, correlations between PM_2.5_ concentration and APR or HR Weibos were all significant and positive for each year during 2017–2021 (as mentioned in Section 3.2.1). Therefore, the spatial distribution of PM_2.5_ concentration and related APR or HR Weibos is examined in the following paragraphs.

**Figure 6:**
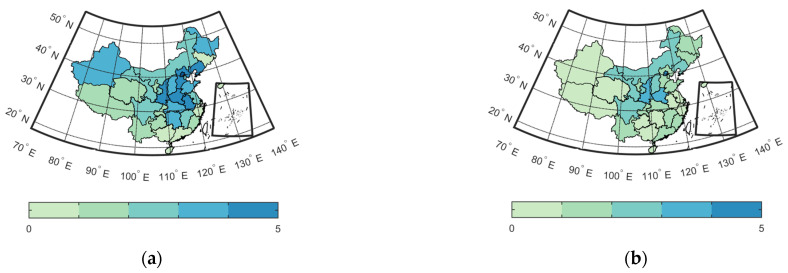
Number of years when correlations between PM_2.5_ concentration and related Weibos were significant and positive for individual PARs for: (**a**) APR Weibos; (**b**) HR Weibos.

Figure 6 shows the number of years (in 2017–2021) during which correlations between PM_2.5_ concentration and related Weibos were significant (*p* < 0.05) and positive (*r* > 0). If a PAR showed a significant and positive correlation for all five years in 2017–2021, the correlation between PM_2.5_ concentration and related Weibos was significant and stable there and residents in the PAR were sensitive to changes in PM_2.5_ concentration during the entire period. However, if a PAR did not have a single year from 2017 to 2021 with a significant and positive correlation between PM_2.5_ concentration and related Weibos, then residents in the particular PAR may not pay attention to air pollution and related health outcomes.

The spatial distribution of correlations between PM_2.5_ concentration and APR Weibos shown in Figure 6a was analogous to the annual average PM_2.5_ concentrations shown in Figure 3d. Beijing and Hebei from the Beijing–Tianjin–Hebei region, Anhui and Henan from Central Plains, and Shanxi from Northwest China were the PARs with more than 4 years of significant and positive correlations between PM_2.5_ concentration and APR Weibos, while the annual average PM_2.5_ concentrations in these PARs were all higher than 44 μg/m^3^. Yunnan and Guizhou from the Yunnan–Guizhou Plateau, Tibet from the Qinghai–Tibet Plateau, and Fujian and Guangdong from southeastern coastal areas were the PARs with less than one year of significant and positive correlations between PM_2.5_ concentration and APR Weibos, and the annual average PM_2.5_ concentrations in these regions were all lower than 27 μg/m^3^. In other words, correlations between PM_2.5_ concentration and APR Weibos were often significant and stable in PARs with poor air quality.

The spatial distribution of correlations between PM_2.5_ concentration and HR Weibos shown in Figure 6b was similar to that of the annual average PM_2.5_ concentration shown in Figure 3d, but there were some differences between them, possibly due to socioeconomic factors. Beijing from the Beijing–Tianjin–Hebei region, Shaanxi from Northwest China, and Henan from Central Plains were the PARs with more than three years of significant and positive correlations between PM_2.5_ concentration and HR Weibos, and the annual average PM_2.5_ concentrations in the PARs were 44.20 μg/m^3^, 48.32 μg/m^3^, and 57.66 μg/m^3^, respectively. However, the PARs with high annual average PM_2.5_ concentrations may not have had significant and stable correlations between PM_2.5_ concentrations and HR Weibos. For instance, the annual average PM_2.5_ concentration in Hebei was 51.62 μg/m^3^, which was extremely high compared with the whole of China, but Hebei had only one year during 2017–2021 with a significant and positive correlation between PM_2.5_ concentration and HR Weibos. In Hebei, GDP per capita was only 48,564 yuan in 2020 (compared with 71,489 yuan in China) and schooling years per capita were 9.35 years in 2020 (compared to 9.50 years in China). Given the underdeveloped economy and slightly lower education levels in Hebei, residents may not be aware of the importance of health issues and therefore may not have posted more HR Weibos when air quality worsened.

#### 3.4.2. Categories of PARs with Different Level of Air Pollution

According to the long-term interim targets 1 and 2 (the annual average PM_2.5_ concentrations of 35 μg/m^3^ and 25 μg/m^3^, respectively) recommended by the WHO global air quality guidelines, 31 PARs in this research were divided into three categories: PARs with good air quality (an annual average PM_2.5_ concentration of lower than 25 μg/m^3^), PARs with moderate air quality (an annual average PM_2.5_ concentration of between 25 and 35 μg/m^3^) and PARs with poor air quality (an annual average PM_2.5_ concentration of higher than 35 μg/m^3^) [26].

**Figure 7:**
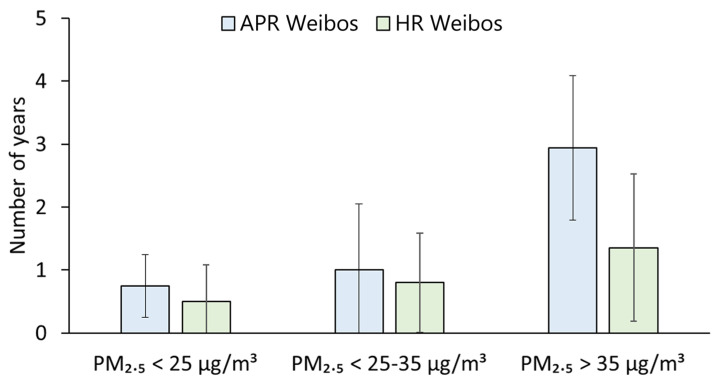
Number of years with significant and positive correlation of PM_2.5_ against APR Weibos and HR Weibos for the three groups of PARs.

Compared with the PARs with moderate or good air quality, PARs with poor air quality had more years when there were significant (*p* < 0.05) and positive (*r* > 0) correlations between PM_2.5_ concentration and APR or HR Weibos (see Figure 7). On average, there were 2.94 years with significant and positive correlations between PM_2.5_ concentration and APR Weibos in the PARs with poor air quality, and there were significant and positive correlations for less than 1 year for PARs with moderate or good air quality. Similarly, the average number of years with significant and positive correlations between PM_2.5_ concentration and HR Weibos in the PARs with poor air quality (1.35 years) was also higher than that in PARs with moderate or good air quality. In other words, PARs with poor air quality had a longer duration when the correlation between PM_2.5_ concentration and APR or HR Weibos was more statistically significant and stable rather than PARs with moderate and good air quality. This is consistent with the trend shown in Figure S2, in which the correlations between PM_2.5_ concentration and related Weibos was stronger in the PARs with poor air quality. These results are similar to those from a previous study that considered three cities from different countries and demonstrated that correlations between PM_2.5_ concentrations and tweets with hashtags related to air pollution increased with increasing PM_2.5_ concentrations. London and Paris, with relatively low PM_2.5_ values, showed lower correlations between PM_2.5_ concentrations and air-pollution-related or health-related tweets, while New Delhi, with the highest pollution level of the three cities, had the strongest correlation between PM_2.5_ concentration and air-pollution-related or health-related tweets [25]. Public concern about air pollution and related health outcomes on social media is likely to rise with increasing air pollution levels in places with poor air quality.

**Figure 8:**
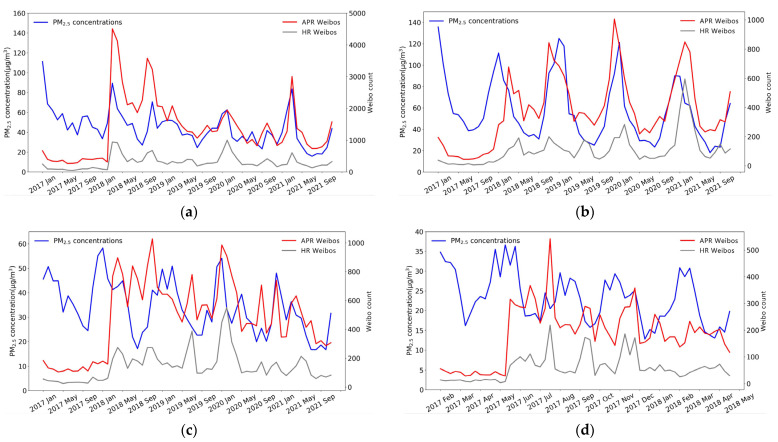
Trends of monthly APR Weibos, monthly HR Weibos, and monthly average PM_2.5_ concentration in 2017–2021: (**a**) Beijing; (**b**) Henan; (**c**) Shanghai; (**d**) Fujian.

The trends of the monthly average PM_2.5_ concentrations and monthly frequency of APR Weibos and HR Weibos are shown for examples of PARs with good (Fujian), moderate (Shanghai), and poor air quality (Beijing and Henan) in Figure 8.

On one hand, in the PARs with poor air quality, trends of PM_2.5_ concentration, APR Weibos, and HR Weibos were consistent, and APR or HR Weibos followed a characteristic seasonal cycle of PM_2.5_ concentration [57]. Figure 8a shows that PM_2.5_ concentration, APR Weibos, and HR Weibos for Beijing all reached their yearly peaks in January 2017, March 2018, February 2020, and March 2021. As the capital of China, Beijing has a dense population (21.89 million in 2020) and the highest GDP per capita (164,889 yuan in 2020) and schooling years per capita (12.21 years in 2020) in China, so poor air quality, as well as socioeconomic factors, made the correlation between PM_2.5_ concentration and APR or HR Weibos strong and stable in Beijing. In contrast, Henan, which also has poor air quality, showed different trends with respect to the posting of APR and HR Weibos. The PM_2.5_ concentration, APR Weibos, and HR Weibos for Henan reached their yearly peaks in January 2017 and January 2020 (Figure 8b). Although PM_2.5_ concentration was extremely high in Henan (the annual average PM_2.5_ concentration was 57.67 μg/m^3^), the GDP per capita of Henan was only 55,435 yuan in 2020 (compared with 71,489 yuan in China), so the correlation between the PM_2.5_ concentration and APR or HR Weibos was not as strong and stable as in Beijing due to the socioeconomic differences between the two locations.

On the other hand, trends of PM_2.5_ concentration, APR Weibos and HR Weibos were not totally consistent in the PARs with moderate and good air quality. In Shanghai, an example PAR with moderate air quality, the monthly trend was only partly consistent since PM_2.5_ concentration and APR Weibos only reached their peak in January 2020 (Figure 8c). Fujian was taken as an example of a PAR with good air quality (Figure 8d), and while its PM_2.5_ concentration followed a characteristic seasonal cycle, the number of posted APR Weibos and HR Weibos fluctuated. Despite the highly developed economies of Shanghai and Fujian (their GDP per capita in 2020 were in the top five in China), the monthly trends of PM_2.5_ concentration, APR Weibos, and HR Weibos were not consistent because Shanghai and Fujian are located in southeastern coastal areas of China with relatively low pollutant concentrations. Residents may not pay much attention to changes in PM_2.5_ concentration because air pollution is not a serious problem there.

### 3.5. Limitations

This study tried to elucidate trends in air pollution and APR and HR Weibos, but there were some limitations in the pre-processing of the Weibo data in our study that can be improved in future studies.

First, the relevance of Weibo as a social media platform may have decreased in the recent years. A large number of air-pollution-related media messages (*n* = 1,559,667) were filtered out by a set of keywords, and a relatively smaller set (30.09%) of individual messages (*n* = 469,340) was retained for further data analysis. With the arrival of newer social media platforms in China, many individuals tend to choose newer platforms instead of Sina Weibo to express personal opinions. WeChat, with 1288 million monthly active users (https://static.www.tencent.com/uploads/2022/05/19/1501a739addd20a382dadeda55b3a7aa.pdf (accessed on 13 August 2022)), has become one of the most popular social media platforms in China [58]. TikTok, launched in 2016 with more than 800 million active users [59], is considered to be the video-sharing platform with the most user-generated content for social communication in China [60,61]. However, WeChat and TikTok do not provide researcher-friendly APIs to directly collect data by keywords or posting time. Therefore, Sina Weibo was the best choice to garner information regarding messages on social media with content related to air pollution and users’ registration places that could be used to conduct a comparison with air pollution data despite it being a less popular social media platform. New platforms such as WeChat and TikTok can be considered in the future to make analyses more comprehensive.

Second, the sentiment analysis of Weibos was not taken into consideration in the current study. The Valence Aware Dictionary for Sentiment Reasoning (VADER), a lexicon and rule-based sentiment classifier for microblogs in English, was used in a previous study that stratified negative and positive tweets related to air pollution and analyzed the correlation between PM_2.5_ concentration and negative tweets in London [33]. The results indicated that negative individual messages, which were separated by a manual qualitative classification method, were found to be more strongly correlated with the AQI than individual messages that included positive content [27]. However, a mature sentiment classifier for microblogs in Chinese is currently unavailable. A processing algorithm to quantify the feelings and attitudes of each Weibo in Chinese may be considered in future research to make analyses more accurate.

## 4. Conclusions

By examining correlations between concentrations of air pollutants (PM_2.5_, PM_10_, CO, NO_2_, O_3_ and SO_2_) and air-pollution-related individual messages (APR) or health-related individual messages (HR) in Sina Weibo in 31 PARs in China during 2017–2021, it was determined that only PM_2.5_ concentration was significantly correlated with APR or HR Weibos for the both entirety of China and individual PARs, and the relationship between PM_2.5_ and APR or HR Weibos remained stable during the study period. Therefore, it can be interpreted that the public is most sensitive to PM_2.5_ concentrations in China. Based on the dataset of Sina Weibo and air pollution that was collected over five years, it is clear from the perspective of temporal variation or spatial distribution that the level of air pollution is always associated with correlations between pollutant concentrations and APR or HR Weibos. Correlations between pollutant concentrations and APR or HR Weibos were found to be stronger if pollutant concentrations were higher in that period or location. In addition to pollutant concentrations, socioeconomic factors can influence APR or HR Weibo postings and further affect the correlation between pollutant concentrations and related Weibos.

Our approach shows that the application of Sina Weibo data can help the government monitor public concern about air pollution and related health outcomes in real time and across the country, as well as enable scientists to survey public response to fluctuations in air pollutant concentrations. This approach of monitoring public concern about air pollution can potentially contribute to the Sustainable Development Goals-UN 2030 agenda [62] since it can indicate how socioeconomic factors affect the perception of air pollution and can thus be useful in proposing measures that can make cities and human settlements sustainable. Moreover, the results showed that the government should pay more attention to the public’s mental health when the concentrations of air pollutants, especially PM_2.5_, increases. When drafting policy or formulating measures for emission control to deal with air pollution, the government may first tackle PM_2.5_ if it incurs similar costs as other air pollutants from the perspective of public concern. In places where residents show less concern about air pollution and related health outcomes on social media, the government should make greater efforts to educate the public about the health effects of air pollution, since the public’s sensitivity to air pollution can be influenced by socioeconomic factors including education.

## Figures and Tables

**Table 1:** Examples of health-related keywords used for data annotation.

Category	Examples of Health-Related Keywords
General Health-Related Words	“健康” (health), “身体” (body), “病” (disease), “生命” (life), and “医院” (hospital)
Body Parts	“肺” (lung), “支气管” (bronchus), “嗓子” (throat), “鼻子” (nose), and “心脏” (heart)
Prevention and Treatment	“口罩” (mask), “疫苗” (vaccine), “药” (medicine), and “抗生素” (antibiotic)
Diseases and Symptoms	Respiratory Tract Infections: “鼻炎” (rhinitis), “咽炎” (pharyngitis), “支气管炎” (bronchitis), “肺炎” (pneumonia), “呼吸” (breath), and “咳” (cough)
	Cancer: “肺癌” (lung cancer), “白血病” (leukemia), and “肿瘤” (tumor)
	Dermatitis and Allergies: “过敏” (allergies), “痘” (pimple), and “疹” (rash)
	Mental Illness: “压力” (pressure), “烦” (upset), and “抑郁” (depression)
	Others: “感染” (infection), “死亡” (death), and “疫情” (epidemic)

**Table 2:** Correlation coefficient between APR and HR Weibos in each PAR during 2017–2021.

PAR	APR Weibos	HR Weibos	Correlation Coefficient	PAR	APR Weibos	HR Weibos	Correlation Coefficient
Anhui	9675	2652	0.786 **	Jiangxi	5867	1639	0.768 **
Beijing	81,749	18,338	0.865 **	Inner Mongolia	4601	1251	0.814 **
Chongqing	7259	2139	0.780 **	Liaoning	13,236	3282	0.861 **
Fujian	11,073	3548	0.854 **	Ningxia	3457	928	0.872 **
Gansu	3961	1057	0.866 **	Qinghai	2093	471	0.670 **
Guangdong	46,827	14,140	0.845 **	Shandong	37,250	7528	0.807 **
Guangxi	6156	1807	0.662 **	Shanxi	9532	2444	0.843 **
Guizhou	4697	988	0.340 **	Shaanxi	16,508	4243	0.941 **
Hainan	2579	678	0.776 **	Shanghai	28,777	8542	0.848 **
Hebei	18,916	4586	0.867 **	Sichuan	22,387	5837	0.869 **
Henan	22,785	6580	0.754 **	Tibet	808	172	0.783 **
Heilongjiang	7034	2137	0.864 **	Tianjin	11,241	2778	0.752 **
Hubei	14,587	3834	0.723 **	Xinjiang	3314	817	0.813 **
Hunan	10,274	2883	0.731 **	Yunnan	5362	1679	0.840 **
Jilin	5612	1547	0.845 **	Zhejiang	24,143	6972	0.822 **
Jiangsu	27,935	7603	0.801 **				

** Pearson correlation coefficient is significant at *p* < 0.01.

**Table 3:** Correlation between pollutant concentrations and APR or HR Weibos in each year of 2017–2021.

Type of Weibo	Year	CO	NO_2_	O_3_	PM_2.5_	PM_10_	SO_2_
APR Weibos	2017	0.150 **	0.426 **	−0.137 **	0.312 **	0.174 **	−0.059
	2018	−0.074	0.292 **	0.043	0.157 **	0.071	−0.196 **
	2019	0.113 *	0.402 **	−0.078	0.315 **	0.206 **	−0.112 *
	2020	0.093	0.267 **	−0.086	0.273 **	0.107 *	−0.195 **
	2021	0.041	0.272 **	−0.064	0.266 **	0.191 **	−0.233 **
HR Weibos	2017	0.124 *	0.402 **	−0.150 **	0.265 **	0.126 **	−0.087
	2018	−0.089	0.302 **	0.039	0.143 **	0.059	−0.202 **
	2019	−0.015	0.209 **	0.040	0.119 *	0.006	−0.194 **
	2020	0.085	0.159 **	−0.081	0.242 **	0.055	−0.183 **
	2021	0.024	0.215 **	−0.038	0.219 **	0.156 **	−0.238 **

** Pearson correlation coefficient is significant at *p* < 0.01, and * Pearson correlation coefficient is significant at *p* < 0.05.

**Table 4:** Correlation between APR or HR Weibos and socioeconomic factors during 2017–2020 ^1^.

	Population	GDP Per Capita	Schooling Years Per Capita	APR Weibos	HR Weibos
Population	1				
GDP per capita	0.060 **	1			
Schooling years per capita	0.001	0.680 **	1		
APR Weibos	0.331 **	0.555 **	0.464 **	1	
HR Weibos	0.354 **	0.533 **	0.434 **	0.901 **	1

^1^ This correlation analysis did not include samples in 2021 since the data on GDP per capita and schooling years per capita of 2021 had not been announced by the national bureau of statistics in China at the time the article was written. Schooling years per capita were calculated for populations of more than 6 years old. The statistics for 2020 were from the national census, and the data for 2017–2019 were based on spot checks that comprised 0.824‰, 0.820‰ and 0.780‰ of the total population, respectively. ** Pearson correlation coefficient is significant at *p* < 0.01.

**Table 5:** Correlation between correlation coefficients of between PM_2.5_ concentrations and APR or HR weibos and socioeconomic factors in 2017–2020 ^1^.

	Population	GDP Per Capita	Schooling Years Per Capita	*r*_1_	*r*_2_
Population	1				
GDP per capita	0.064	1			
Schooling years per capita	0.001	0.680 **	1		
*r*_1_	0.071	0.082	0.206 *	1	
*r*_2_	0.046	0.053	0.243 **	0.814 **	1

^1^ This correlation analyses did not include samples in 2021 since the data on GDP per capita and schooling years per capita of 2021 had not been announced by the national bureau of statistics in China at the time the article was written. Schooling years per capita were calculated for populations of more than 6 years old. The statistics for 2020 were from the national census, and the data for 2017–2019 were based on spot checks that comprised 0.824‰, 0.820‰ and 0.780‰ of the total population, respectively. ** Pearson correlation coefficient is significant at *p* < 0.01. * Pearson correlation coefficient is significant at *p* < 0.05.

## Data Availability

The data presented in this study are available on request from the corresponding author. The data are not publicly available due to the privacy of participants.

## Supplementary Materials

The following supporting information can be downloaded at: https://www.mdpi.com/article/10.3390/ijerph192316115/s1. Figure S1: Word clouds of (a) air-pollution-related keywords and (b) health-related keywords in individual messages on Sina Weibo; Figure S2: Scatter plots between correlation coefficients and the pollutant annual average concentrations in 2017–2021 for the different air pollutants: (a) NO_2_ concentration vs. APR Weibos; (b) NO_2_ concentration vs. HR Weibos; (c) PM_2.5_ concentration vs. APR Weibos; (d) PM_2.5_ concentration vs. HR Weibos; (e) PM_10_ concentration vs. APR Weibos; (f) PM_10_ concentration vs. HR Weibos.
