# Supplementary material for: Public Concern about Air Pollution and Related Health Outcomes on Social Media in China: An Analysis of Data from Sina Weibo (Chinese Twitter) and Air Monitoring Stations
Source: Int J Environ Res Public Health. 2022 Dec 1;19(23):16115. doi: 10.3390/ijerph192316115 (PMC9740218; doi:10.3390/ijerph192316115)
Supplement: Supplementary file 1 [file ijerph-19-16115-s001.zip › ijerph-1996701-supplementary.pdf]

Public Concern on Air Pollution and Related Health Outcomes on Social Media in China: An Analysis of Data from Sina Weibo (Chinese Twitter) and Air Monitoring Stations

Binbin Ye [1,*], Padmaja Krishnan [2] and Shiguo Jia [3,4,5]

[1] College of Chinese Language and Culture, Jinan University, Guangzhou 510610, China

[2] Division of Engineering, New York University Abu Dhabi, Abu Dhabi P.O. Box 129188, United Arab Emirates

[3] School of Atmospheric Sciences, Sun Yat-sen University and Southern Marine Science and Engineering Guangdong Laboratory (Zhuhai), Zhuhai 519082, China

[4] Guangdong Provincial Field Observation and Research Station for Climate Environment and Air Quality Change in the Pearl River Estuary, Guangzhou 510275, China

[5] Guangdong Province Key Laboratory for Climate Change and Natural Disaster Studies, Sun Yat-sen University, Guangzhou 510275, China

Supplementary Material

Contains 2 pages, 2 figures

* Corresponding author.

E-mail address: yebinbin@hwy.jnu.edu.cn

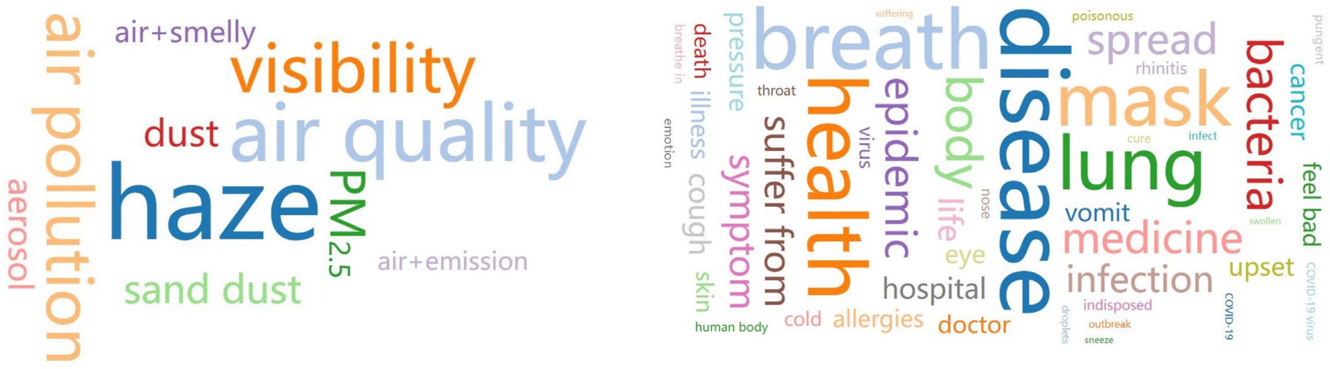

(**a**) (**b**)

Figure S1. Word clouds of (**a**) air-pollution-related keywords and (**b**) health-related keywords in individual messages on Sina Weibo. (**b**) did not contain all the health-related words in this study because some words are too small to read due to their relatively low frequency of occurrence. Health-related words that showed up more than 1500 times in APR weibos were selected in the word clouds of health-related keywords in individual messages on Sina Weibo

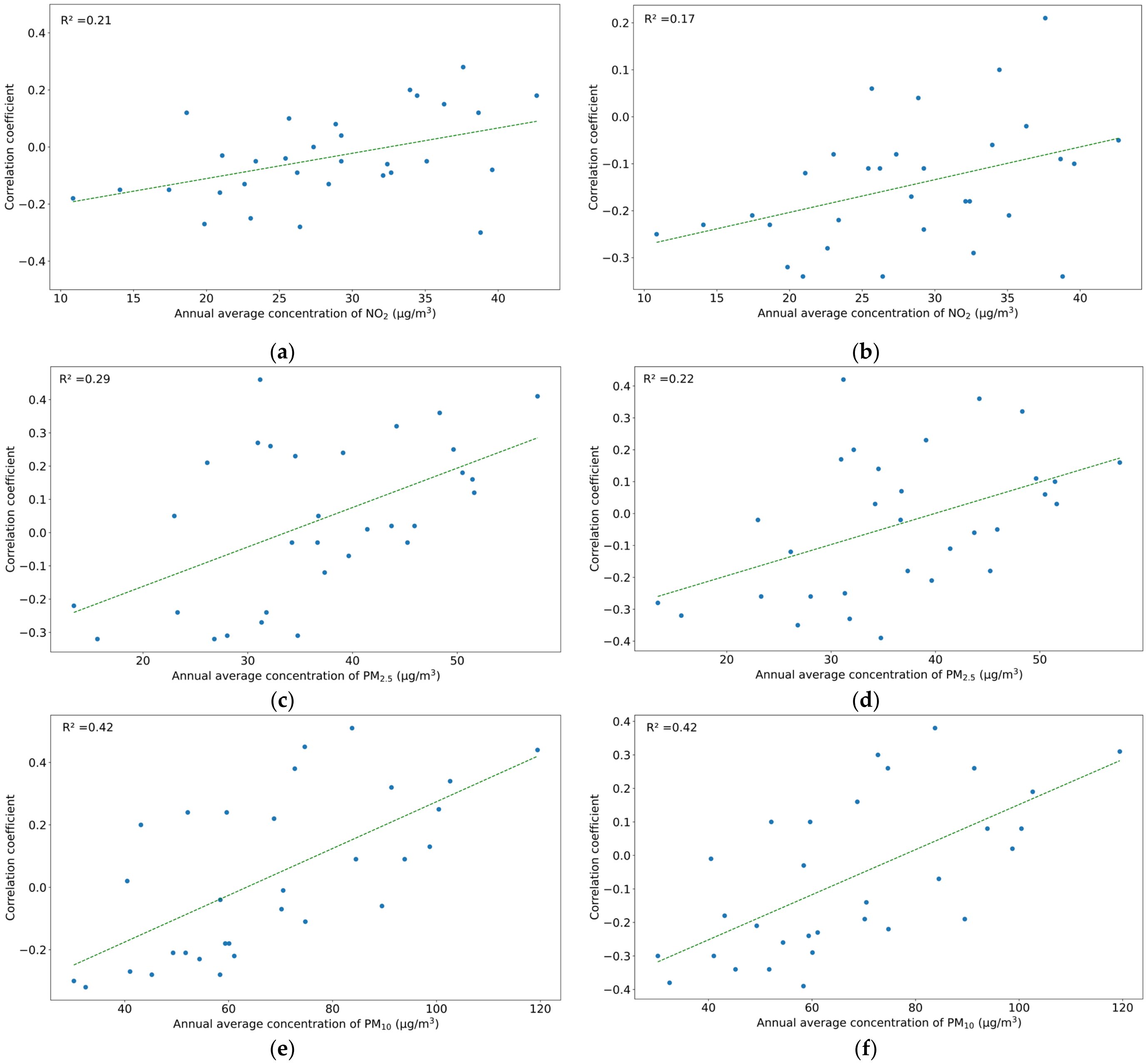

Figure S2. Scatter plots between correlation coefficients and the pollutant annual average concentrations in 2017-2021 for the different air pollutants: (**a**) NO_2 concentration vs. APR weibos; (**b**) NO_2 concentration vs. HR weibos; (**c**) $PM_{2.5}$ concentration vs. APR weibos; (**d**) $PM_{2.5}$ concentration vs. HR weibos; (**e**) PM_{10} concentration vs. APR weibos; (**f**) PM_{10} concentration vs. HR weibos.
